# Adhesive Joint Stiffness in the Aspect of FEM Modelling

**DOI:** 10.3390/ma12233911

**Published:** 2019-11-26

**Authors:** Kamil Anasiewicz, Józef Kuczmaszewski

**Affiliations:** Department of Mechanical Engineering, Lublin University of Technology, 20-388 Lublin, Poland

**Keywords:** apparent Young’s modulus, nanoindentation, adhesive joint zones, changes in adhesive properties

## Abstract

The paper presents the results of nanoindentation testing, carried out along the thickness of the adhesive joint joining sheets of aluminum alloy. The purpose of the tests was to determine changes in the Young’s modulus in the joint resulting from the active impact of the joined aluminum alloy sheets on the adhesive during curing of the adhesive bond. Structural changes that take place during curing of the joint, especially in the boundary zone, can have a significant impact on the adhesive properties and consequently, on the adhesive joint strength. The Young’s modulus of the adhesive (Ek) in the joint assumes variable values as the distance from the connections changes. This phenomenon is called the apparent Young’s modulus. The problem is to define the size of the boundary zone in which the value of Ek significantly differs from the value in the so-called core. Based on the obtained results of experimental tests, a numerical model was built taking into account the observed differences in the properties of the joint material. The stress distribution in the adhesive joint, single-lap connection with the three-zone adhesive joint, was analyzed in comparison to the classical numerical model in which adhesive in the adhesive joint is treated as isotropic in terms of rigidity.

## 1. Introduction

Due to the rapid development of adhesive technology, which is implemented in many industries, it is especially important to effectively forecast the strength of adhesive-bonded structures. It is not always possible to carry out destructive tests, hence the development of methods for forecasting the strength of adhesive joints based on theoretical and especially numerical models, which can significantly reduce the costs of preparing production. In adhesive joints, destruction usually occurs in the adhesive or cohesive zone of the adhesive joint, less often in the adhesive zone of the connectors. Bonding effects are influenced by many factors of a technological and construction nature. Obtaining an effective (with a high degree of convergence with the results of destructive tests) forecast load capacity, based on FEM simulation, depends on the proper definition of boundary conditions, including material properties [1,2]. Based on the authors’ own research [3], it is known that the Young’s modulus of the adhesive, defined in the tensile test of the “dumbbell sample” of the material cured in the form of a cast material, may significantly differ from its value in the boundary zone of the adhesive joint. The purpose of the tests is to determine the size of the boundary zone, to determine the physical Ek values for selected adhesives and to analyze the results of the “sensitivity” of the FEM model to include Ek changes in the 3-zone model compared to the model with isotropic stiffness of the joint along its thickness. In accordance with the standards set out in the standards, dumbbell samples are made to determine the basic material properties of the adhesive. Samples are usually prepared in silicone moulds by casting a previously prepared adhesive composition. After curing under defined conditions, the samples are removed from the moulds, milled to specific sizes, and then subjected to destruction on a testing machine. Such research allows us to determine, among others, the values of Young’s modulus, Poisson’s ratio or yield strength of the tested material. Strength testing in the axial tensile test allows average results for hardened adhesive material to be obtained. Analyzing the presented test methodology, the question arises about the curing conditions of the dumbbell sample compared to the adhesive joint cured in direct contact with the surface of the bonded material. Doubts related to the adoption of the same properties of the adhesive material, identical to the adhesive material in the form of a dumbbell sample, are even more intensified when taking into account the relatively small thickness of the adhesive joint, oscillating within 0.1 mm or less. Previous studies have shown differences in the properties of the adhesive material in relation to the properties of the adhesive material cured in the form of a dumbbell sample [3].

The scope of application of adhesive joints covers especially such areas of technology as the aviation industry, automotive industry, construction, electrical engineering and electronics and others. This means that such connections are subject to high operational requirements, especially those related to the need to ensure the safe use of this type of structure [4,5]. Checking the strength of the adhesive joint in the single overlap, double overlap or metal thick adherend and derivative configurations requires multiple series of samples to determine the strength of the tested joint [6,7]. Such research requires a lot of time and money. Therefore, actions are required to increase the accuracy of modelling of adhesive joints using the finite element method by examining the consequences of phenomena occurring in the adhesive joint during curing. 

The adhesive in the liquid state, in contact with the joined material, especially the metal, is subject to the strong influence of the field of physisorption and chemisorption forces at the boundary of the phases. In addition, due to the large heat capacity of the metal, it strongly receives heat from the cured adhesive, and this reaction is usually exothermic. This may have a direct effect in the form of differentiation of the physical properties of the adhesive, including crosslinking density and some “ordering” of the structure in chemosetting adhesives in the area located in the zone of the boundary of the phases compared to the material in the center of the joint. The crosslink density of epoxy resin has a direct impact on the adhesive properties and affects its rigidity and strength. In contrast to the dumbbell sample, the adhesive thickens in thin joints in the presence of energetic impact of rigid connection elements and different thermal curing conditions in the boundary and middle of the joint. This leads to a complicated state of stress in the loaded joint as a consequence of structural changes in the cross-section of the joint [8,9]. The stress–strain ratio in the joint cross-section in the boundary zone is defined as the apparent Young’s modulus E’. In the theoretical case, when the joint is perfectly bound by connections whose deformation is equal to zero, the ratio between the apparent and real Young’s modulus is expressed by formula 1 [10,11]:

(1) $$\frac{E^{\prime }}{E}=\frac{\left(1-\upsilon \right)}{\left(1+\upsilon \right)\left(1-2\upsilon \right)}$$

where: υ–adhesive Poisson’s ratio, E–Young’s modulus in the middle zone of the joint.

**Figure 1:**
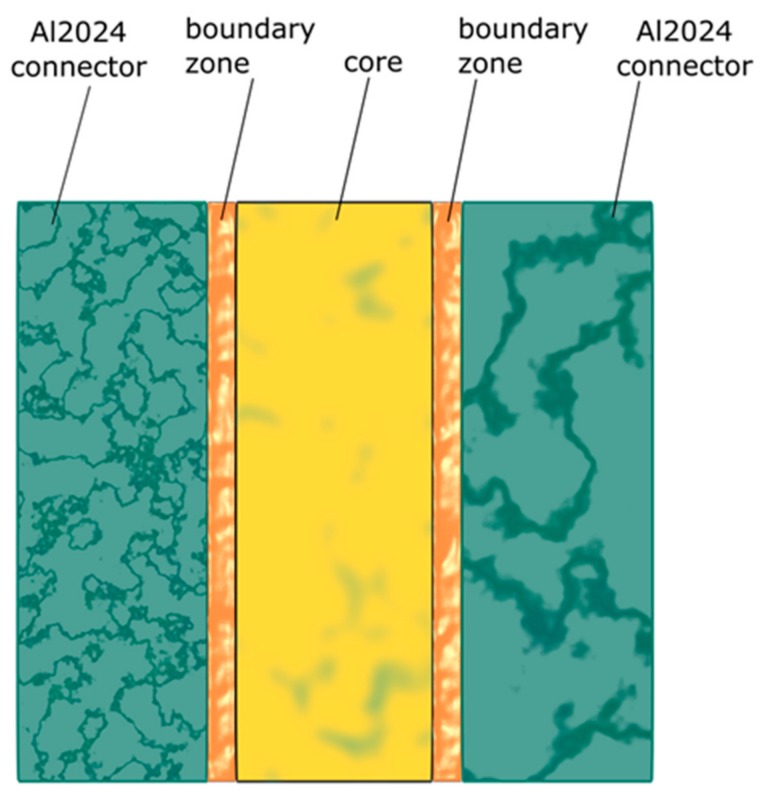
Proposed division of the joint into zones.

For the purposes of the article, the zones (Figure 1) with potentially different properties were distinguished in the adhesive joint. The joint was divided into two boundary zones in direct contact with the bonded material and a zone of the joint core located in the middle, between the boundary zones. In addition, separate transition zones are located between the boundary areas and the joint core.

Division of the joint into zones is arbitrary and results from differences in material properties between the zones. The width of the zones is reflected in the depth of deposition of Young’s modulus changes in the joint. The boundary between zones is arbitrary, and the change in material properties between zones is continuous.

**Figure 2:**
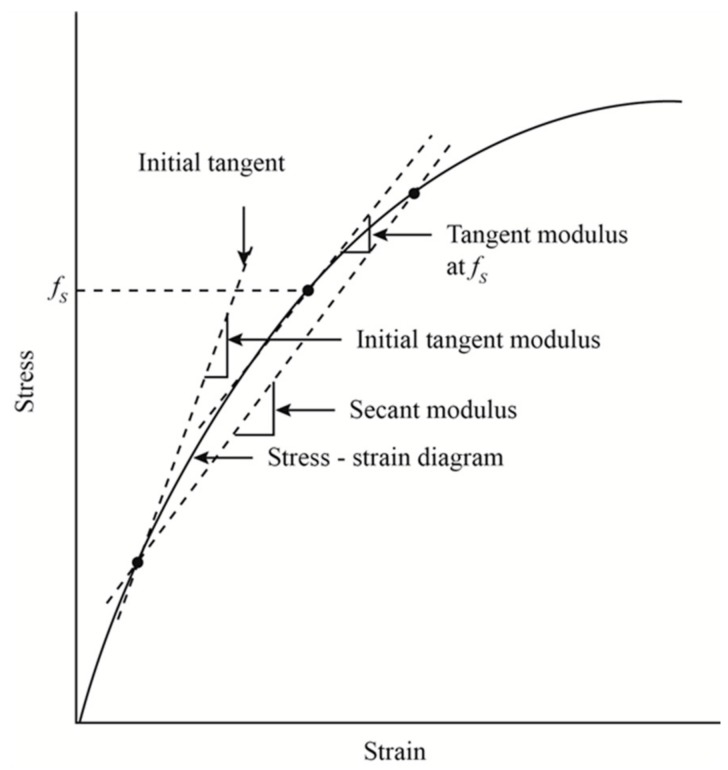
Graphic interpretation of Young’s module types.

This problem is not widely covered in the literature. For the needs of FEM numerical simulation, in addition to the already mentioned classical approach, sometimes the secant Young modulus is adopted [10]. Its essence is shown in Figure 2, and its value is determined on the basis of characteristics. 

The phenomenon known as the apparent Young’s modulus is known [10,11,12], but the Ek = f(gk) characteristics are not defined. The factors determining the size of the boundary zone are also unknown.

## 2. Materials and Methods

**Figure 3:**
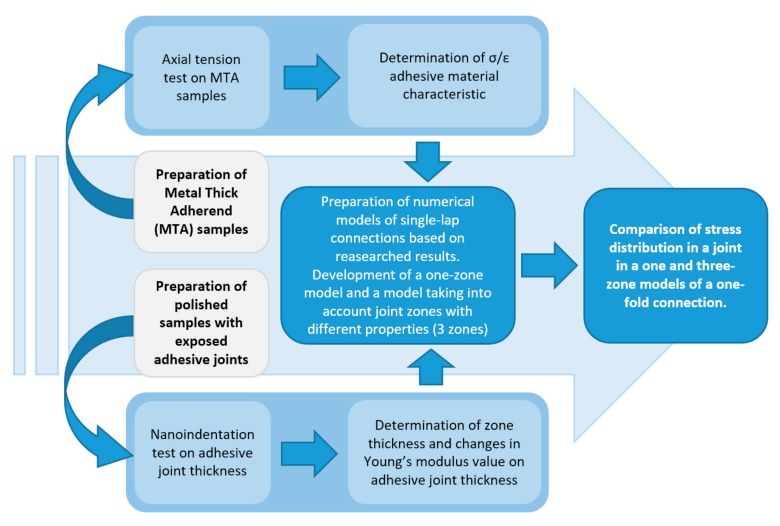
Block diagram of tests carried out.

In order to determine the changes in the properties of hardened joint on the thickness of the adhesive joint and the impact of these changes on the adhesive connection, tests were carried out according to the scheme shown in Figure 3. In the first stage, the samples were made of two glued EN AW 2024-T3 aluminum alloy plates. The sample was glued with 3M Scotch Weld EC-9323 B/A. It is a two-component structural epoxy adhesive in the form of a paste. The adhesive has very good adhesion to various materials, including metals, glass, ceramics and plastics, including GFRP and CFRP composites. After curing, it provides high resistance to shear and breaking forces in a wide temperature range. It also has good resistance to harsh environmental conditions and chemicals [13]. The nominal value of the Young’s modulus of the adhesive declared by the manufacturer is 2.4 GPa. The adhesive was mixed in the proportions (100:27 by mass) recommended by the manufacturer. Before bonding, the panels were cleaned and prepared using the Sol-Gel method, applying 3M Surface Pre-treatment AC-131. The adhesive was evenly applied to both surfaces of the plates. The joint was cured in a vacuum bag under a pressure of 0.1 Mpa for a period of 24 h. After 7 days, the bonded sheets were pre-cut with a water-abrasive stream to expose the adhesive joint and then to samples of dimensions 10 × 4 mm with the joint exposed.

The surface of the samples to be tested was initially levelled by grinding on grinding wheels using intensive cooling. Further processing was carried out on abrasive papers, starting with coarse grain paper (180, 240 grit size) and ending with papers with the smallest grain size (1000, 1200 grit size). During grinding the conditions provided helped to minimize thermal impact on the sample. After grinding, the samples were polished mechanically, on horizontally positioned rotary discs lined with felt, covered with an aqueous suspension of Al_2_O_3_. Polishing continued until a mirror-like surface without scratches. The polished sample was washed in water and ethyl alcohol, and then dried in a stream of air. 

**Figure 4:**
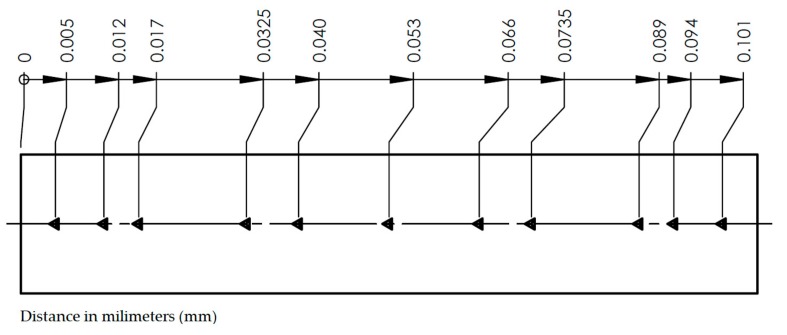
Arrangement of indentations on the thickness of the adhesive joint.

The study was performed using the CSM ultra nanoindentation instruments. The device permits measuring microhardness and calculating the hardness and Young’s modulus using the Olivier–Pharr method with high accuracy. Nanohardness can be defined as a measure of material resistance during insertion of an indenter perpendicular to its surface, with defined geometry and defined material properties [14,15]. The test was carried out on the thickness of the adhesive joint using a reference head, allowing indenter cavities to be maintained in relation to the material surface. Indentations were made at eleven points at fixed distances from each other. The scheme of the distribution of measurement points is presented in Figure 4. There were 10 repetitions for each point in different places in the adhesive joint. Nanoindentations were performed using a diamond indenter with Berkovich geometry to a set depth of 800 nm. The first of the measurements was made as close as possible to the interface of the aluminum alloy–adhesive, at a distance of 0.005 mm. Due to the requirement to maintain an appropriate distance between subsequent prints, in order to eliminate the impact of subsequent prints on each other, they were moved relative to each other instead of placing them in line perpendicular to the connected connections.

Based on the results of the experiment, a numerical model was prepared using ABAQUS software [16]. The aim of the study is to compare the state of stress in the adhesive joint in the FEM model, taking into account the differences in material properties over the thickness of the real joint and a model of the joint with homogeneous material properties. In order to define the significance of differences, the model was built solely taking into account changed values of Young’s modulus obtained in the study. The model does not take into account the viscoelastic characteristics of the adhesive and its yield strength for a more complete determination of changes in the level of stress in the joint. A single-lap sample in 2D representation in two variants, with dimensions in accordance with ASTM D1002, was selected for verification. In the first variant, the 0.1 mm thick adhesive joint was presented as one zone, with the same Young’s modulus value in the entire adhesive joint. In the second variant, the 0.1 mm thick joint is divided into 3 zones—2 boundary zones 0.015 mm thick each and the joint core 0.07 mm thick. Boundary zones were assigned a higher value of Young’s modulus. The connection elements are modelled as aluminum alloy sheets. In the area of the end of the lap, the division into finite elements was concentrated. Each of the adhesive layers in the second variant of the model is divided into a minimum of 3 finite elements. As a result, the size of a single finite element in the boundary zone is 0.005 mm. The finite element type was modelled as solid. The model has been given restrictions that reflect the tension test in the jaws of the testing machine. One end of the connection has been given a defined force value. The obtained results were compared in terms of differences in values and stress distribution in the adhesive joint. Table 1 shows material constants used in the FEM model.

To determine additional simulation parameters, metal–thick–adherend (MTA) samples made of aluminum alloy EN AN 2043-T3 were prepared. The connections were joined with 3M Scotch Weld EC-9323 B/A adhesive and prepared in accordance with ASTM D5656 [7]. Samples were made under conditions equivalent to those used in the nanoindentation study.

**Figure 5:**
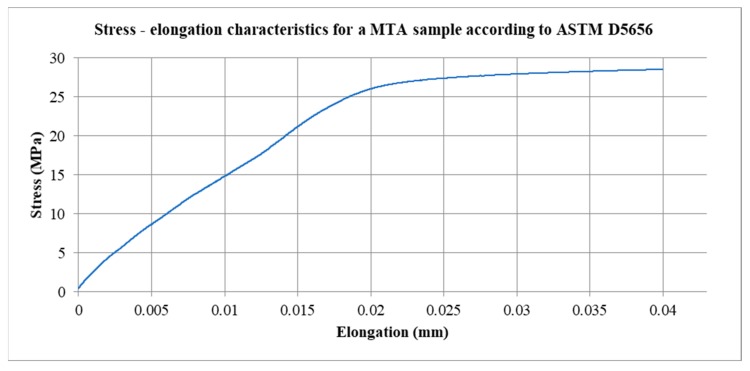
Stress–elongation characteristics for an MTA sample according to ASTM D5656.

The prepared samples were tested on a Zwick Roell Z150 testing machine using an averaging extensometer for ASTM D5656 Adhesive Lap Shear Tests-Model 4013. From the 5 tests, the stress–elongation characteristics were determined. As a defined value of force applied to the connection plate in the FEM model, a force of 8400 N was adopted. This force corresponds to the limit value of displacement in the elastic limit −0.02 mm for the MTA sample. Figure 5 presents a graph from the MTA sample tensile test.

## 3. Results

**Figure 6:**
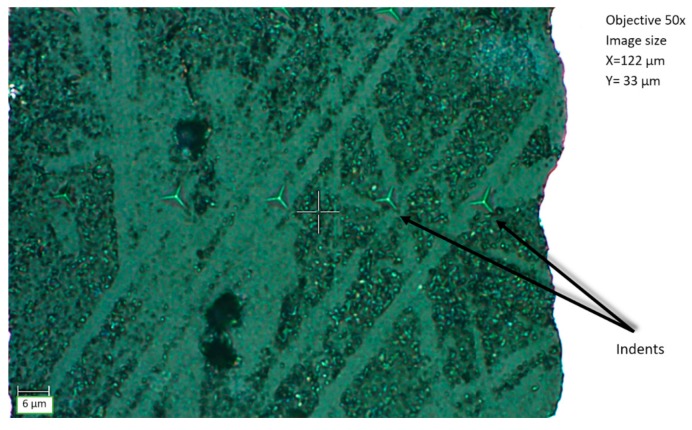
A series of indentations in the adhesive material.

The results of the nanoindentation test from 11 measuring points were developed in the form of hardness diagrams and Young’s modulus values depending on the distance from the joint edge. The values were additionally averaged for one side of the joint. An example of the adhesive surface with performed indentations is shown in Figure 6.

**Figure 7:**
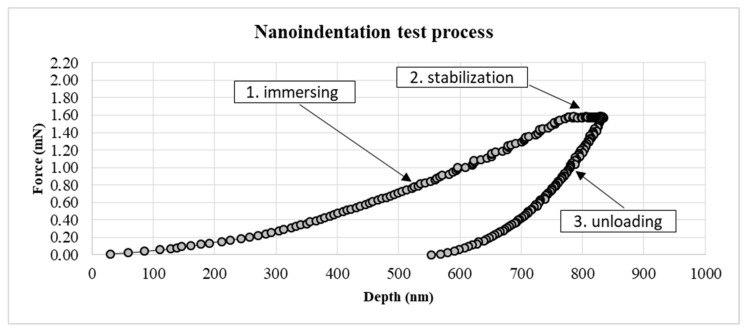
Examination of the adhesive joint with a Berkovich indenter.

Figure 7 shows the course of a single indentation. The graph shows the dependence of the measured force on the depth of the depression. The chart shows three main curves presented as a set of points. The immerse (loading) curve shows the force required to immerse the indenter to a precisely defined depth. The stabilization curve indicates when the indenter reaches maximum depth. The unloading curve shows the exit of the indenter from the material and the material response to indentation. Owing to the presented characteristics and precise measurement, it is possible to calculate the hardness and Young’s modulus of the tested material.

**Figure 8:**
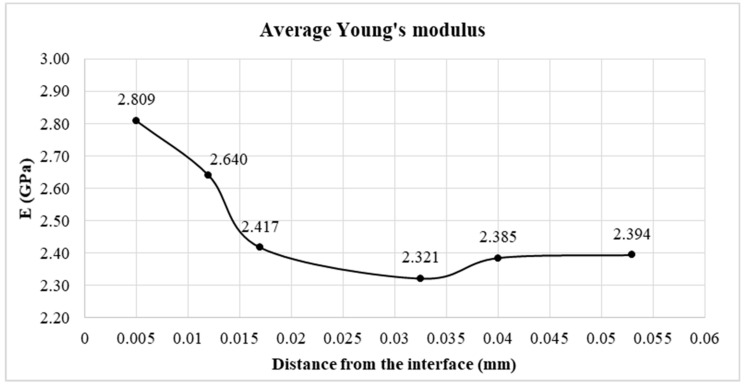
The distribution of the Young’s modulus along the thickness of the adhesive joint.

Figure 8 presents the average values of Young’s modulus over the thickness of the adhesive joint. The measurement was carried out across the entire thickness of the glue joint equal to 0.107 mm, however, the results were additionally averaged by presenting them only on half the thickness of the measured joint. According to the previously presented proposal, the graph, and thus also the adhesive joint, can be divided into 3 main zones. Joint material situated the closest to the interface has a higher average Young’s modulus value than the rest of the joint. The range of changes that cause an increase in the Young’s modulus value reaches a depth of about 0.015 mm. The average Young’s modulus in this zone, the boundary zone, is 2.72 GPa. Then a decrease in Young’s modulus in the transition zone is observed. It is worth noting the decrease in the Young’s modulus of the adhesive made at the 0.0325 mm point. This is a slight decrease compared to the basic Young’s modulus value represented by the joint core. The average value of Young’s modulus in the transition zones and joint core is 2.38 GPa. The difference in the average value of Young’s modulus of the boundary zones in relation to the combined transition zone and the joint core is 14.5%. The value of the Young’s modulus value at the core equal to the Young’s modulus value declared by the adhesive manufacturer confirms the correctness of the test. The presented values are the average of 20 measurements at a set distance from the joint edge. Extreme values were discarded. It should be noted, however, that the technical considerations of the measurement, and more precisely the limited possibility of the indenter approach to the connector material, mean that the actual Ek value in the zone immediately adjacent to the aluminum alloy is probably even higher. It seems that for FEM analysis in the boundary zone, the maximum value Ek should be taken, not the average value. This will be verified in further studies.

**Figure 9:**
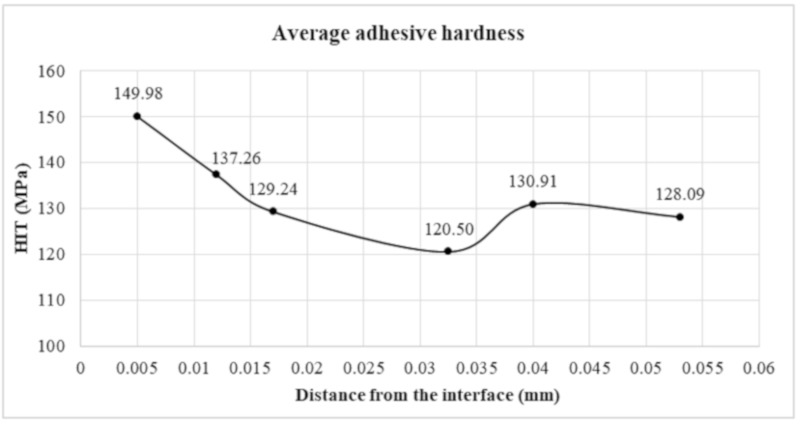
Hardness distribution along the thickness of the adhesive joint according to the Olivier–Pharr method.

Figure 9 presents the average adhesive hardness over the thickness of the adhesive joint. As in the case of Young’s modulus results, the average hardness is shown for half the thickness of the adhesive joint. Hardness is expressed in MPa according to the Olivier–Pharr method.

The hardness of the joint material in direct contact with the connection is the highest. Then a reduction in hardness was observed, which stabilizes within the joint core. Again, a decrease in hardness is visible at a distance of 0.0325 mm from the interface. This can be attributed to the difference in crosslinking density of the adhesive material. There is a fairly clear correlation between hardness in the boundary zone and the value of Ek. Table 2 presents summary values from conducted tests.

**Figure 10:**
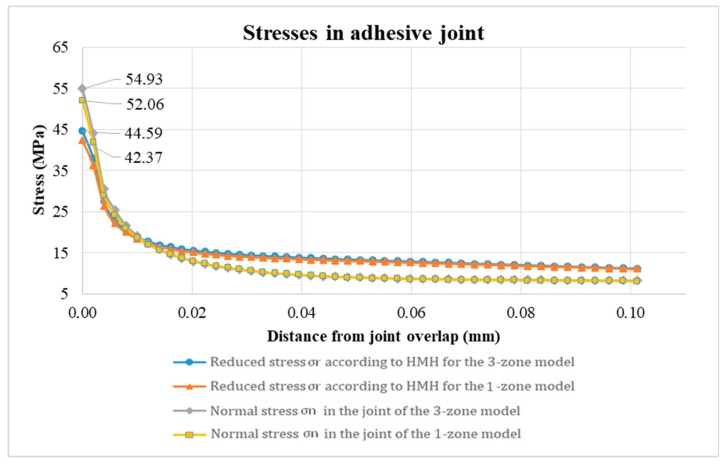
Comparison of reduced stresses according to von Mises yield criterion (HMH) and normal stresses along the edge of the adhesive joint.

Figure 10 presents a comparison of reduced stress distribution and normal stress along the overlap in both FEM models. The increase in stress in the boundary zone can have a direct effect in initiating the destruction of the adhesive connection. In the graph, the distance along the overlap is limited to 0.1 mm to emphasize changes in stress levels in this critical place for the strength of the adhesive connection.

The tensions assume the expected values in a characteristic way, increasing at the ends of the overlap. The waveforms in both models are almost identical except for the maximum stress values reduced at the end of the overlap in the zone in direct contact with the connector.

## 4. Discussion

The research allowed to formulate the following major conclusions:

1. A decrease in the Young’s modulus of the adhesive was observed along with increasing distance from the adhesive zone of the bonded material and the adhesive;
2. On the basis of the presented tests, it is possible to separate the zones in the glue joint differing in values of the Young’s modulus into two boundary zones and the joint core. The increase in Young’s modulus in the boundary zone reaches 18%. It should be noted, however, that at a distance closer than 0.005 mm the value of Young’s modulus may be higher;
3. The nominal value of the Young’s modulus, declared by the manufacturer, was only achieved within the joint core. The thickness of the adhesive layer characterized by the changed Young’s modulus value was determined. It is about 0.015 mm measured from the edge of the metal–adhesive joint interface. A change in the Young’s modulus value within the boundary zone resulted in an increase in reduced stress in the joint, in particular in the boundary zone. The highest tensions are achieved at the end of the overlap, i.e. in the place where the initiation of damage to the adhesive joint can be expected;
4. The application of the FEM model, taking into account additional boundary zones of the adhesive joint, influenced the highest reduced stress and normal stress according to the HMH hypothesis. It achieved a value 5.5% higher in the model including three zones in the adhesive joint compared to the traditional model treating the entire volume of adhesive as isotropic;
5. The obtained results indicate that the hypothesis of the largest normal stress reflects the conditions of destruction of overlap joints quite well.

Increased tensions at the ends of the overlap of the adhesive connection have a decisive impact on the strength of the single-lap connection, because it is the normal stress at the ends of the overlap that destroy the connection. Taking into account changes in the adhesive joint occurring during curing allows for more accurate prediction of the strength of the joint. 

## Figures and Tables

**Table 1:** The material constants used in the FEM model.

Item	Materials	Young’s Module (MPa)	Poisson’s Ratio (-)
1	Core zone of 3M adhesive joint	2400	0.4
2	Boundary zone of 3M adhesive joint	2800	0.4
3	Connectors EN AW 2024-T3	72,000	0.33

**Table 2:** Summary of Young’s modulus and hardness values depending on the distance from the joint edge.

No.	Distance from the Joint Edge (mm)	Change of Young’s Modulus			Change in Hardness		
		Young’s Joint Module (GPa)	Standard Deviations (%)	Change of Young’s Modulus in Relation to the Joint Core (%)	Hardness HIT (MPa)	Standard Deviations (%)	Change in Hardness in Relation to the Joint Core (%)
**1**	0.005	2.81	3.9%	18%	149.98	7.6%	17%
2	0.012	2.64	4.8%	10%	137.26	10.0%	7%
3	0.017	2.42	2.4%	1%	129.24	5.0%	1%
4	0.0325	2.32	3.4%	-3%	120.50	13.2%	-6%
5	0.04	2.39	2.9%	0%	130.91	5.5%	2%
6	0.053	2.39	3.9%	0%	128.09	7.6%	0%
